# NIR‐I Activated Orthogonal NIR‐IIb/c Emissions in a Lanthanide‐Doped Nanoparticle for Fluorescence Imaging and Information Encryption

**DOI:** 10.1002/advs.202408097

**Published:** 2024-09-30

**Authors:** Qiqing Li, Yuanping Huang, Haoyu Zhu, Yaqi Zhu, Yuexi Yi, Xiaodan Li, Haoran Chen, Bin Li, Dabing Li, Yulei Chang

**Affiliations:** ^1^ Key Laboratory of Luminescence Science and Technology Chinese Academy of Sciences & State Key Laboratory of Luminescence Science and Applications Changchun Institute of Optics Fine Mechanics and Physics Chinese Academy of Sciences Changchun Jilin 130033 China; ^2^ State Key Laboratory on Integrated Optoelectronics Key Laboratory of Advanced Gas Sensors College of Electronic Science and Engineering Jilin University Changchun Jilin 130033 China; ^3^ Department of Respiratory Medicine The First Hospital of Jilin University Changchun Jilin 130033 China; ^4^ Northeast Normal University Changchun Jilin 130033 China

**Keywords:** lanthanides, NIR‐II, encryption, orthogonal emission, ratiometric fluorescence imaging

## Abstract

Applying the orthogonal principle for distinguishable second near‐infrared (NIR‐II) emissions has brought new dimensions for ratio fluorescence imaging (RFI) detection and information encryption, deepening the tissue detection depth and improving signal‐to‐noise ratio and information security. However, the orthogonal NIR‐II emissions underlying these advanced optical applications have been reported only in heterogeneous structures and mixtures, limiting their practicality and potential impact. Herein, NIR‐I‐activated orthogonal NIR‐IIb/c (1530/1825 nm) emissions nanoparticles (ONNPs) are developed by spatially separated doping of Tm^3+^ and Er^3+^ emitter upon switching 808 and 980 nm excitations. RFI techniques and orthogonal NIR‐II emission ONNPs are used to demonstrate vessel depth detection based on wavelength‐dependent optical attenuation properties in tissue. The superiority of the optical coding and encoding process in a 4 × 1 binary matrix is demonstrated for anticounterfeiting and decryption imaging of quick‐response (QR) code for information storage. The research progress of this NIR‐II orthogonal emissions probe will drive the development of biomedical sensing, imaging safety, and future biophotonics technologies.

## Graphical abstract

The orthogonal NIR‐IIb/c nanoparticles are designed by core‐shell‐shell‐shell structured Li‐based fluoride, producing independent emissions at 1825 and 1530 nm by switching the 808/980 nm excitation. According to their photo‐switchable NIR‐IIb/c emissions, this work demonstrates their great potential in ratiometric fluorescence imaging and optical information security.

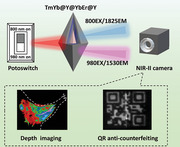

## 1 Introduction

Fluorescence imaging has been considered one of the promising imaging strategies in information encryption and biological applications due to its readability, real‐time, exceptional spatiotemporal resolution, and non‐invasive characteristics.^[^ ^1^ ^]^ Compared to visible (400–700 nm) and first near‐infrared light (NIR‐I, 700–1000 nm), second near‐infrared (NIR‐II, 1000–2000 nm) light can decrease the background luminescence and increase the penetration depth and signal‐to‐noise ratio efficiently simultaneously in biological tissue, due to the benefit of reduced light scattering and tissue autofluorescence.^[^ ^2^ ^]^ Especially, given the absorption intensity of water, tissue scattering, and detector range in the NIR‐II region, the NIR‐II region beyond 1500 nm exhibits better imaging performance, which has also been defined as two subwindows: NIR‐IIb (1500–1700 nm) and NIR‐IIc (1700–2000 nm). In addition, due to the invisible spectral range of NIR‐II fluorescence, it has become an ideal choice for encryption information and anticounterfeiting.^[^ ^3^ ^]^

Lanthanide‐doped nanoparticles (LnNPs) have exhibited extraordinary luminescence features characterized by high photostability, narrowband emission, larger anti‐/Stokes shift, and long luminescence decay time.^[^ ^4^ ^]^ Benefiting the rich energy levels of lanthanides, LnNPs can be excited by multiple NIR incident lights and produce various NIR‐II emissions, which provide more fluxes and dimensions for NIR‐II multiplexed imaging,^[^ ^5^ ^]^ encoding^[^ ^6^ ^]^ and anticounterfeiting.^[^ ^7^ ^]^ For example, several lanthanides have been reported to produce NIR‐II emission through self‐/sensitization strategies, e.g., Er^3+^, Nd^3+^, Ho^3+^ and Tm^3+^ doped LnNPs produce 1530, 1064/1310, 1185/2000, 1450/1825 nm emissions, respectively,^[^ ^5^, ^8^ ^]^ in response to excitation at different wavelengths, rendering them highly suitable for diverse NIR‐II imaging applications.^[^ ^9^ ^]^ However, traditional fluorescence imaging technologies have considerable limitations, such as limited in obtaining the depth information of vessels based on the fluorescent intensities of agents despite enabling high‐resolution 2D imaging of blood vessels.^[^ ^10^ ^]^ Besides, traditional probes for fluorescent imaging lack the tunability to allow encoding in a single material, causing low‐security levels and easy cracking. Fortunately, orthogonal luminescence (OL) materials can solve the above problems. For instance, ratiometric fluorescence imaging (RFI) techniques can utilize two emission bands from one OL probe to obtain the depth information of blood vessels with a self‐calibrated reference signal independent of concentration.^[^ ^11^ ^]^ Traditionally, using two emission bands of two types of dyes, the relationship between fluorescence ratio and target concentration is obtained, while errors can easily arise due to the concentration difference between the two different probes. Fortunately, the fluorescence ratio between two emission bands of orthogonal luminescent materials is independent of the concentration, making it more accurate. Moreover, achieving a balanced or switched dual emission of OL nanoparticles can alter the emission color or wavelength, which is also desirable for information encryption applications. Especially, their long lifetimes, sharp emission peaks, stability, and multiplexing capabilities make them a powerful tool for encoding and storing information with high fidelity and robustness. These facts indicate that the orthogonal NIR‐II emissions have greater advantages in RFI and optical coding, but underlying these advanced optical applications have been reported only in heterogeneous structures and mixtures, limiting their practicality and potential impact.^[^ ^12^ ^]^ Due to the structural designability of LnNPs, the strategy of orthogonal luminescence in one single nanoparticle can be achieved by comprising inert spacing layers, where the interlayer energy transfer can be selectively blocked to avoid crosstalk, which is more stable and reliable.

**Figure 1:**
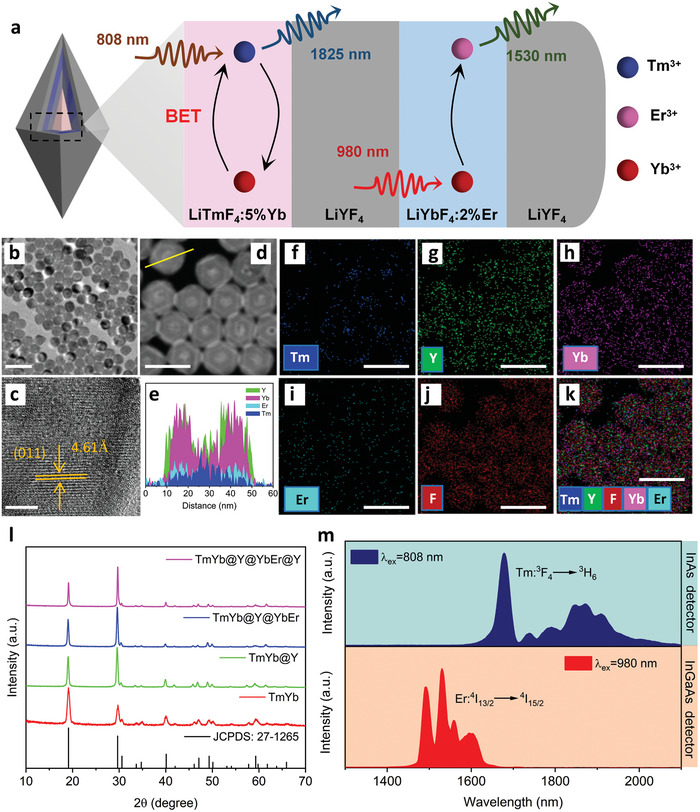
a) Schematic illustration of LiTmF_4_:5%Yb@LiYF_4_@LiYbF_4_:2%Er@LiYF_4_ nanostructure for 1530/1825 nm orthogonal downshifting emissions under 808/980 nm excitations. b) TEM and c) high‐resolution TEM image and d) HAADF‐STEM images of LiTmF_4_:Yb@LiYF_4_@LiYbF_4_:Er@LiYF_4_ NPs, and corresponding e) linear scanning and f–k) elemental mappings of Tm, Y, Yb, Er, and F. l) XRD patterns of samples in (a) and standard card of tetragonal LiYF_4_. m) DSL spectra of the nanoparticles under 808 and 980 nm excitations, respectively. Scale Bar: b) 100; c) 5; f–k) 50 nm.

Herein, we present orthogonal NIR‐IIb/c downshifting luminescence (DSL) emissions at 1530/1825 nm in novel core‐shell structured LiTmF_4_:Yb@LiYF_4_@LiYbF_4_:Er@LiYF_4_ nanoparticles (ONNPs) under dual NIR‐switching excitations (808 and 980 nm) (**Figure** **1**a). Specifically, taking advantage of the blocking effect of the inert LiYF_4_ layer, the activators of Tm^3+^ and Er^3+^ were finely separated, and their energy interaction was suppressed, achieving the orthogonal NIR‐II emissions under different excitations. As a proof‐of‐concept, we demonstrated their utility for potential RFI in response to the depth of blood vessels under 808 and 980 nm excitation. Besides, we demonstrate the optical coding and encoding process in a 4 × 1 binary matrix using four different LnNPs and decryption imaging of quick‐response (QR) code. The unique photoluminescent properties of these nanoparticles represent a critical advance in many applications involving the construction of optical storage devices for information storage and encryption, bioimaging, and sensing.

## 2 Results and Discussion

A modified coprecipitation and thermal decomposition method were used to prepare LiTmF_4_:5%Yb@LiYF_4_@LiYbF_4_:2%Er@LiYF_4_ core‐multishell orthogonal NIR emission nanoparticles (Figure 1a,b). Compared to oxide and sulfide, alkali fluoride has low phonon energy. Besides, the tetragonal phase of LiLnF_4_ host has lower crystal symmetry than the cubic phase of NaYF_4_ which could improve the luminescence of doped lanthanide ions.

**Figure 2:**
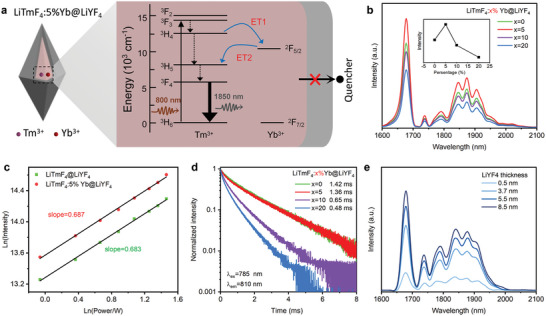
a) Schematic illustration of LiTmF_4_:Yb@LiYF_4_ nanostructure for 1825 nm downshifting emissions under 808 nm excitations. b) Downshifting emission spectra of a) sample with different Yb^3+^ doping concentrations (0, 5, 10, and 20%). The emission intensities were normalized by the absorption of samples at 808 nm. c) Power‐dependent downshifting luminescence intensities of LiTmF_4_:Yb@LiYF_4_ and LiTmF_4_@LiYF_4_. d) Time‐resolved luminescence decay curves of b) samples under 810 nm under 785 nm excitation. e) Downshifting emission spectra of a) sample with different inert shell thicknesses.

Transmission electron microscopy (TEM) images showed a uniform morphology with an increase in nanoparticle size after the epitaxial growth of each shell layer during the synthetic procedure (Figure S1, Supporting Information). The morphology of all particles is rhombic with long and short axes. The size of nanoparticles increased from 23.1 ± 1.5 nm to 48.2 ± 1.8 nm for the long axis and from 15.0 ± 1.2 nm to 47.8 ± 1.5 nm for the short axis, respectively. The HAADF‐STEM image showed the lattice fringe of (011) planes with a d‐spacing of 4.61 Å. The final core@shell@shell@shell nanostructure is also confirmed by the EDS line scanning and element mapping, showing contrast in the distributions of different lanthanide elements (Figure 1e–k). X‐ray diffraction (XRD) patterns confirmed the tetragonal phase of as‐obtained nanoparticles according to standard card JCPDS#27‐1265 (**Figure** **2**l).

**Figure 3:**
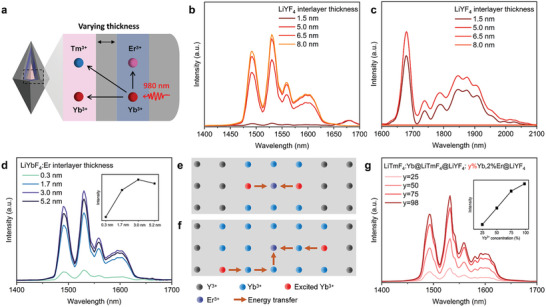
a) Schematic illustration of interlayer thickness‐tunable ONNPs nanostructure and possible energy transfer process under 980 nm excitations. b,c) Downshifting emission spectra of a) samples with a different thickness of LiYF_4_ interlayer under 980 nm excitations. d) Downshifting emission spectra of ONNPs with different layer thicknesses of LiYbF_4_:Er interlayer under 980 nm excitations. The emission intensities are normalized by the absorption of Yb^3+^ at 980 nm. e,f) Schematic illustration showing proposed energy transfer from Yb^3+^ to Tm^3+^ in Yb‐sublattice of varying thickness of LiYbF_4_:Er interlayer. g) DSL spectra of ONNPs with different Yb^3+^ concentrations (25, 50, 75, and 98%) in LiYbF_4_:Er interlayer under 980 nm excitations.

The related orthogonal DSL emission profiles are shown in Figure 1m. In terms of luminescence, the structure mainly consists of two parts, namely, the Tm^3+^ emission band in the core and the Er^3+^ emission band in the shell. Upon 808 nm laser irradiation, it only effectively excites Tm^3+^ activators in the core region instead of Er^3+^ in the shell layer due to a low Er^3+^ doping level, realizing the peaked NIR‐II emission at 1825 nm (Figure 1m, upper chart). When photoswitch turn to 980 nm excitation, the Yb host sensitized LiYbF_4_:Er layer harvests the 980 nm energy and then preferentially transfers the energy to adjacent Er^3+^ activators to promote the DSL at 1530 nm (Figure). Next, we optimized the structure of ONNPs to obtain the target luminescence characteristics. First of all, to enhance the 1825 nm emission, a small amount of Yb^3+^ was introduced to modulate the ^3^F_4_ state of Tm^3+^ upon 808 nm excitation from Yb^3+^ to Tm^3+^ in the LiTmF_4_:Yb core. As shown in Figure 2b and Figure S2 (Supporting Information), the NIR emission in LiTmF_4_:x%Yb@LiYF_4_ is enhanced by the presence of Yb^3+^ and reaches a maximum at 5 mol% doping level. This remarkable increase in the downshifting intensity might be ascribed to the BET from Yb^3+^ to Tm^3+^, an additional population of the ^3^F_4_ state to reduce the dependence on the pumping power of the 808 nm laser (Figure 2a). Moreover, power‐dependent investigation of the nanoparticles indicates that both LiTmF_4_@LiYF_4_ and LiTmF_4_:Yb@LiYF_4_ nanoparticles have similar slope values (Figure 2c), were measured to be 0.683 and 0.687, respectively, indicating that both are one photon DSL. To verify the influence on the power density dependence of the downshifting luminescence, a representative five levels simulation model is set up with energy transfer and BET to address the luminescence kinetics of Tm^3+^ and Yb^3+^ (discussed in Section S1, Supporting Information). In this simulation model, two energy levels of Yb^3+^ and five levels of Tm^3+^ are mainly involved. Tm^3+^ is excited to the T_4_ level (^3^F_2_) by harvesting the incident photons and then transitioning to the T_3_ (^3^F_3_) level via nonradiative relaxation. When Yb^3+^ is close to Tm^3+^, Tm^3+^ transfers energy to the ground state of the Yb^3+^, pumping the Yb^3+^ to an excited state (Y_1_, ^2^F_5/2_). The Yb^3+^ in the excited state pumps Tm^3+^ to an excited state (T_2_, ^3^H_5_) by further BET. Finally, Tm^3+^ relaxed to the ^3^F_4_ level and then transitioned to the ground state, emitting a NIR photon (λ = 1825 nm). This luminescence enhancement mechanism is attributed to energy transfer and reverse energy transfer, which has also been reported in Er^3+^‐Yb^3+^ co‐doped materials.^[^ ^13^ ^]^ The rate equations describe the steady‐state populations of the relevant energy levels, as shown in Section S2 (Supporting Information). The population of excited states reflects the luminescence intensity. By solving the equations, we find that the population of the ^3^F_4_ level is enhanced due to the Yb^3+^ doping. The enhancement through populating the ^3^F_4_ level is determined by the energy transfer rate (*ω*) and back energy transfer rate (*ω_b_*):

(1) $$\frac{\alpha_{1}\beta_{2}}{\left(\alpha_{1}+\beta_{1}\right)\left(\alpha_{2}+\beta_{2}\right)}\cdot \frac{\omega \omega_{b}}{\xi_{1}+\upsilon_{1}+\omega_{b}}T_{3}$$

where ξ_1_ and $\upsilon_{1}$ represent radiative and nonradiative rates of ^2^F_5/2_ level (Yb^3+^), respectively. However, the Yb^3+^ doping does not change the power density‐dependent relationship of emission intensities of Tm^3+^, which agrees with the experimental results (Figure 2c). Moreover, to investigate the luminescence mechanism, the time‐resolved emission spectroscopy from the ^3^H_4_ state of Tm^3+^ was recorded, as shown in **Figure** **3**d. It can be found that the lifetime of the ^3^H_4_ state decreases when the concentration of Yb^3+^ increases, indicating the energy transfers from the ^3^H_4_ (Tm^3+^) state to the ^2^F_5/2_ (Yb^3+^) state. We further find that the emission intensity increases with the increasing inert shell thickness from 1.5 ± 0.2 nm to 8.0 ± 1.2 nm in the long axis (Figure 3e and Figure S3, Supporting Information). It shows that the thicker the LiYF_4_ interlayer, the brighter the downshifting emission spectra of LiTmF_4_:5%Yb@LiYF_4_ at 1825 under 808 nm excitation because the thicker inert shell can effectively suppress the surface quenching effect.

**Figure 4:**
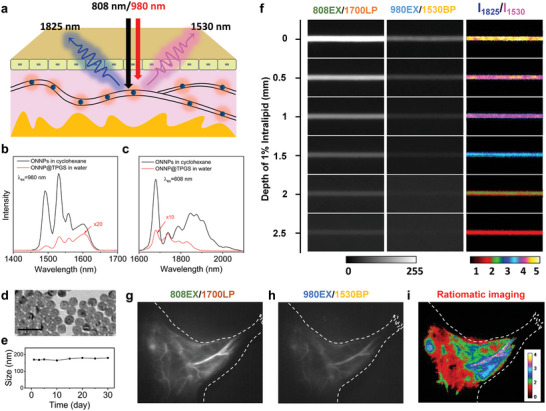
a) Schematic illustration of the ONNP@TPGS probe flowing in the blood vessels. b,c) DSL spectra of ONNPs in cyclohexane and ONNP@TPGS in water under 808 and 980 nm excitation, respectively. d) TEM images and e) stability of hydrophilic ONNP@TPGS probes over time. f) NIR‐II luminescent and ratiometric images of a capillary containing ONNPs ‐based probe covered with different thicknesses of 1 wt% intralipid under 808 or 980 nm excitation. NIR‐II luminescent images of the mouse's right hind were i.v. injected with ONNP‐based probes, including different channels: g)NIR‐IIc (1700 LP: > 1700 nm), h)NIR‐IIb (1530 BP:1524–1536 nm), and i)corresponding ratiometric images.

Furthermore, to block the interactions between different lanthanide emitters, an inert LiYF_4_ interlayer is a compulsory requirement between the YbTm and YbEr co‐doping layers. Thus, the samples with a thickness‐tunable inert LiYF_4_ interlayer between the core and the YbEr layer were synthesized (i.e., LiTmF_4_:5%Yb@LiYF_4_@LiYbF_4_:2%Er@LiYF_4_ NPs), using the LiTmF_4_:Yb@LiYF_4_ nanoparticle as the core (Figure S3, Supporting Information). Their DSL spectra at single 980 nm excitations are shown in Figure 3b,c. Compared to the sample without the inert LiYF_4_ interlayer, when the thickness of the inert interlayer increases, the intensity of Tm^3+^ emission at 1825 nm decreases, but the Er^3+^ emission at 1530 nm increases under 980 nm excitation, same as the upconversion emissions (Figure S4, Supporting Information). This is because the energy transfer from Yb to Tm is blocked and more energy flows toward Er^3+^ ions. The Tm^3+^ emissions are effectively minimized, and the Er^3+^ emission becomes dominant when the thickness of the LiYF_4_ interlayer reaches 6.5 nm in the long axis in length. On the other hand, two possible energy loss channels exist: 1)Tm^3+^→Yb^3+^ and 2)Tm^3+^→Er^3+^, resulting in the quenching of Tm^3+^ DSL. Upon 808 nm excitation, the Tm^3+^ emission at 1825 nm is gradually enhanced by increasing the thickness of the interlayer because of the abatement of the quenching process. Furthermore, the effect of LiYbF_4_:2%Er interlayer thickness on the DSL of Er^3+^ is investigated. Interestingly, it is not that the thicker the YbEr layer, the stronger the DSL of Er^3+^ (Figure 3d and Figure S4, Supporting Information). This phenomenon was also observed in Wang et al.’s work.^[^ ^14^ ^]^ The depletion of excitation energy of Yb^3+^ in thicker layers may be dominantly ascribed to long‐distance energy migration, which increases the number of defects accessible to the Yb sublattice. The detailed possible energy transfer processes are shown in **Figure** **4**e,f. Besides, the concentration of Yb^3+^ ions in the YbEr layer is also an essential factor in determining the emission of ONNPs under 980 nm excitation because it closely relates to the photon‐blocking effect by harvesting the 980 nm photons. Thus, LiTmF_4_:5%Yb@LiYF_4_@LiYF_4_:x%Yb,2%Er@LiYF_4_ (x = 25%, 50%, 75%, 98%) NPs were prepared, and their DSL spectra at NIR region were shown in Figure 4f. It is found that the higher the Yb^3+^ concentration, the stronger the Er^3+^ emission. This is because the higher doping concentration of Yb^3+^ causes the shorter energy transfer distance from Yb^3+^ to Er^3+^, resulting in more efficiency in absorbing and utilizing the 980 nm pumping energy.^[^ ^15^ ^]^ Finally, 98% of Yb^3+^ content is adopted in the YbEr layer.

These ONNPs are practicable for application in luminescence bioimaging. We present as a proof‐of‐concept for the NIR‐II RFI. Owing to the different scattering and absorption of NIR‐II photons in biological tissues, the quenching process of NIR photons varies with the different wavelengths (Figure 4a). Theoretically, the fluorescence signal intensity of a nanoprobe is determined by many factors, for instance, temperature and concentration, while the fluorescence intensity ratio (*R*) is only determined by the depth of nanoprobes under the fixed excitation power of 808 and 980 nm (Section S3, Supporting Information):

(2) $$\ln R\;=C-\left[\Delta \alpha_{1}+\Delta \alpha_{2}\right]l$$

That is to say, the sensitivity of ln *R* to *l* is determined by Δα_1_ and Δα_2_, which represent the different optical absorbance of excitation wavelength and emission wavelength, respectively. Traditionally, the luminescent nanoprobe with multiple emission bands can also be used for ratiometric fluorescence imaging. However, it is only pumped by one excitation and the sensitivity of fluorescence ratio to depth is determined by the optical absorbance of its emission wavelength, which is lower than that of orthogonal luminescent materials.

Traditionally, the luminescent nanoprobe with multiple emission bands also can be used for ratiometric fluorescence imaging. However, it is only pumped by one excitation and the sensitivity of fluorescence ratio to depth is determined by the optical absorbance of its emission wavelength, which is lower than that of orthogonal luminescent materials. Therefore, using an MCT camera and different filters (1530 BP: 1524–1536 nm band‐pass; 1700LP: 1700 nm long‐pass), we first performed a simulation study in 1% intralipid to investigate the tissue depth dependence of ratiometric fluorescence imaging. The ONNPs were functionalized with D‐alpha‐tocopheryl poly (ethylene glycol 1000) succinate and encapsulated into the hydrophobic layer of micelles (ONNP@TPGS) through hydrophobic and hydrophobic interactions. Interestingly, the emission profiles and intensities of ONNP@TPGS in water are different from ONNPs in cyclohexane (Figure 4b,c). Owing to the different absorption coefficients of solvents, the emissions at 1530 and 1680 nm of ONNP@TPGS were ≈180 and ≈37 times weaker than that of ONNPs, respectively. The TEM images have shown no obvious aggregation of ONNPs after functionalization (Figure 4d). The hydrodynamic diameter of ONNP@TPGS probe was 168 ± 2.1 nm according to the dynamic light scattering (DLS) result (Figure 4e) and the MTT result shows the good biocompatibility of ONNP@TPGS below 200 µg mL^−1^ (Figure S6, Supporting Information).

**Figure 5:**
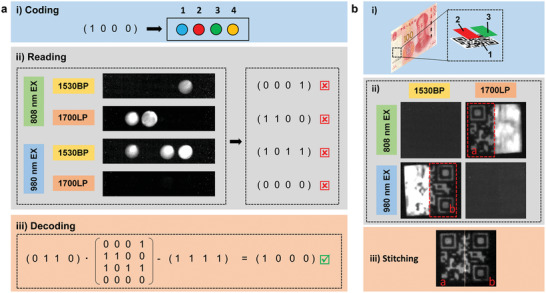
a) Schematic of the binary array using four kinds of NIR nanoparticles and decoding using an MCT camera. b) Schematic of information decoding of a quick‐response code using a NIR camera. The code was prepared using three kinds of inks. ink1: ONNPs; ink2:LiTmF_4_:Yb@LiYF_4_; ink3: LiYbF_4_:Er@LiYF_4_; ink4: LiErF_4_@LiYF_4_.

A capillary filled with ONNP@TPGS probes immersed in intralipid solution generated 1825 and 1530 nm ratiometric fluorescence signals under 808 and 980 nm excitation, respectively (Figure 4f). A plot of fluorescence intensity ratio of 1825/1530 nm fluorescence signal shows that the quenching process displays exponential decay occurring at a depth range of 0–2.5 mm (Figure S5, Supporting Information). Encouraged by the above results, the fluorescence intensity ratiometic imaging in vivo was performed. The ONNP@TPGS probes were delivered through the tail vein injection to light up the blood vessels of the mouse in the right hind leg. Clear and non‐invasive fluorescence imaging of leg blood vessels under the irradiation of 808 nm is depicted in **Figure** **5**g,h, respectively. However, according to the fluorescence intensities, the depth information of blood vessels cannot be obtained from a single image alone. Fortunately, from the 1825/1530 nm fluorescence ratiometric signals for RFI technology, we can realize the vessel profiles in the depth range of 0.5–2.5 mm (Figure 4i). In contrast, due to the low penetration depth of visible and short‐wave infrared light in biological tissues, fluorescence ratio imaging cannot achieve vascular imaging beyond a depth of 1.5 mm (Figure S8, Supporting Information). According to the above results, the ONNP@TPGS is a promising candidate for ratiometric fluorescence imaging. Advanced information encoding and encryption are also investigated to extend further the application of the orthogonal NIR‐IIb/c luminescent performance of ONNPs. As shown in Figure 5a–i, a 4 × 1 binary matrix (1 0 0 0) is encrypted by four different lanthanide‐doped NPs, which can produce different emissions under two‐mode excitation (λ_ex_ = 808 and 980 nm), respectively. Using an MCT camera and different filters, four 4 × 1 binary matrices are recorded from 4 channels, and it cannot read out the accurate information from a single channel alone (Figure 5a–ii). Nevertheless, a 4 × 4 binary matrix was obtained from the above four 4 × 1 binary matrices and we decrypt the actual information from the 4 × 4 binary matrix and the key matrix (0 1 1 0) (Figure 5a–iii). Furthermore, these lanthanide‐doped ONNPs are assembled into a quick‐response (QR) code covered with LiYbF_4_:Er@LiYF_4_ and LiTmF_4_:Yb@LiYF_4_ nanoparticles for information storage and security. As shown in Figure 5b, only half the QR pattern can be read out using a single channel or a single excitation due to the confusing signal. In this case, the whole QR code can be edited from the two patterns (“a” and “b”).

## 3 Conclusion

In summary, we have successfully synthesized lanthanide‐doped core‐multilayer LiTmF_4_:Yb@LiYF_4_@LiYbF_4_:Er@LiYF_4_ NPs with orthogonal NIR luminescent performance. The emission at 1530 nm only contributes to Er^3+^ activators under 980 nm excitation, while the emission at 1825 nm only comes from Tm^3+^ activators under 808 nm excitation. Doping Yb^3+^ sensitizers can further enhance the emission of Tm^3+^ activators due to the energy transfer (from Tm^3+^ to Yb^3+^) and the back energy transfer (from Yb^3+^ to Tm^3+^). Using a sandwich multilayer structure, the excitation energy is confined in the Yb^3+^ sub‐lattice, encouraging the Er^3+^ emission. Furthermore, based on the absorption competition process, the ONNP@TPGS can be used as ratiometric nanoprobes for depth imaging of blood vessels in vivo. The different channels of fluorescence images not only show the 2D distribution of vessels but also provide their depth information. In addition, the ONNPs are employed for binary matrix encryption and QR coding. This unique optical property enables these ONNPs to display excellent potential for advanced information encryption and storage. We believe such a design of ONNPs will inspire further research on developing orthogonal fluorescent probes for diverse applications.

## Conflict of Interest

The authors declare no conflict of interest.

## Supporting information



Supporting Information

## Data Availability

The data that support the findings of this study are available from the corresponding author upon reasonable request.
