# Supplementary material for: NIR‐I Activated Orthogonal NIR‐IIb/c Emissions in a Lanthanide‐Doped Nanoparticle for Fluorescence Imaging and Information Encryption
Source: Adv Sci (Weinh). 2024 Sep 30;11(44):2408097. doi: 10.1002/advs.202408097 (PMC11600275; doi:10.1002/advs.202408097)
Supplement: Supplementary file 1 — Supporting Information [file ADVS-11-2408097-s001.docx]

**Supporting Information**

NIR-I activated orthogonal NIR-IIb/c emissions in a lanthanide-doped nanoparticle for fluorescence imaging and information encryption

Qiqing Li^[a,b]^, Yuanping Huang^[c]^, Haoyu Zhu^[a,d]^, Yaqi Zhu^[a,d]^, Yuexi Yi^[a,d]^, Xiaodan Li^[c]^, Haoran Chen^[a]^, Bin Li^[a]^, Dabing Li^[a]^ and Yulei Chang*^[a]^

[a] Q. Li , H. Zhu, Y. Zhu, Y. Yi, H. Chen, B. Li, D. Li and Y. Chang
Key Laboratory of Luminescence Science and Technology , Chinese Academy of Sciences & State Key Laboratory of Luminescence Science and Applications, Changchun Institute of Optics, Fine Mechanics and Physics, Chinese Academy of Sciences
130033 Changchun, Jilin(China)
E-mail: yuleichang@ciomp.ac.cn

[b] Q. Li
State Key Laboratory on Integrated Optoelectronics, Key Laboratory of Advanced Gas Sensors, College of Electronic Science and Engineering, Jilin University
130033 Changchun, Jilin(China)

[c] Y.Huang and X. Li
Department of Respiratory Medicine, the First Hospital of Jilin University
130033 Changchun, Jilin(China)

[d] H. Zhu, Y. Zhu and Y. Yi
Northeast Normal University
130033 Changchun, Jilin(China)

1. **Experimental section**

**1.1. Chemicals**

TmCl_3_·6H_2_O(99.9%), YbCl_3_·6H_2_O(99.9%), oleic acid (90%) (OA) and 1-octadecene (90%) (ODE) were all purchased from Sigma-Aldrich. Er_2_O_3_(99.9%), Yb_2_O_3_(99.9%), Y_2_O_3_ (99.9%), NaOH, NH_4_F and trifluoroacetic acid were obtained from Aladin. Ethanol and cyclohexane were purchased from Beijing Chemical Works. All other chemical reagents were of analytical grade and were used directly without further purification.

**1.2. Synthesis of LiTmF_4_:x%Yb core NPs**

LiTmF_4_:x%Yb nanoparticles were synthesized based on a previously reported method^[[1](#bib1)]^. TmCl_3_·6H_2_O (1-x mmol) and YbCl_3_·6H_2_O(x mol) were added to OA (6 mL) and ODE (15 mL). The mixture was heated at 150 ℃ under argon protection for 30 min and then cooled to room temperature. NH_4_F (4 mmol) and LiOH·H_2_O (2.5 mmol) were dissolved in methanol (5 mL) and added to the three-necked flask. Afterward, the solution was heated to 70 °C to remove the methanol. Then, the solution was heated to 300 °C and kept for 1.5 h. Finally, the solution was cooled to room temperature and precipitated using ethanol. The solid was collected by centrifugation and dispersed in 10 mL of hexane.

**1.3. Synthesis of LiTmF_4_:Yb@LiYF_4_@LiYbF_4_:Er@LiYF_4_ core-shell NPs**

The shell coating procedure of LiTmF_4_:Yb@LiYF_4_@LiYbF_4_:Er@LiYF_4_ core-shell nanoparticles was executed by a layer-by-layer method. Firstly, the shell precursor was prepared in advance. Specifically, Y_2_O_3_ (1 mmol) and trifluoroacetic acid (0.5 g) were added to a 50 mL flask containing OA (5 mL) and ODE (5 mL). The mixture was heated at 100 ℃ for 30 min and then became a transparent solution. The LiYF_4_ shell precursor was cooled down to room temperature for use. The synthetic procedure for inner LiYbF_4_:Er and outer LiYF_4_ shell precursor was identical to LiYF_4_ except for using different lanthanide oxides.

Then 2 mL of cyclohexane containing 0.2 mmol LiTmF_4_:x%Yb nanocrystals was added to OA (5 mL) and ODE (5 mL). Then, the solution was kept at 70 °C for 30 min to remove hexane. After that, the solution was heated to 300 °C under an argon atmosphere. The three kinds of shell precursor were injected into the solution one by one at a rate of 0.02 mL/min. Finally, the solution was kept at 300 °C for 45 min and then cooled to room temperature. The LiTmF_4_:Yb@LiYF_4_@LiYbF_4_:Er@LiYF_4_ core@shell nanocrystals were precipitated with 10 mL of ethanol, collected by centrifugation, and dispersed in 10 mL of hexane.

**1.4. Synthesis of LiYbF_4_:Er@LiYF_4_, LiTmF_4_:Yb@LiYF_4_ and LiErF_4_@LiYF_4_ core-shell NPs**

The synthetic procedure for LiYbF_4_:Er@LiYF_4_, LiTmF_4_:Yb@LiYF_4_ and LiErF_4_@LiYF_4_ NPs were similar to that of LiYF_4_ except for the use of different raw lanthanide materials.

**1.5. Characterizations**

The transmission electron microscopy (TEM) measurements were carried out using a JEM-2100F electron microscope operating at 200 KV. X-ray diffraction (XRD) measurements were performed with a Bruker D8-advance X-ray diffractometer (XRD) with Cu Kα radiation (λ=1.5418 Å). The spectra were recorded with an FLS980 spectrometer from Edinburgh Instruments. Luminescence decay curve were recorded by a Hamamatsu R9110 PMT single photon counting system and a single exponential fitting model was employed to fit the obtained luminescence lifetime lines, and the fitting formula is $y=y_{0}+A\cdot e^{-\frac{X}{t}}$. Luminescence digital photographs were recorded with a ZephIR 2.5 HgCdTe (MCT) camera (Photon etc).

**1.6. Fluorescence ratiometric imaging**

Fluorescence imaging was achieved using an MCT camera. ONNPs were modified with D-alpha-tocopheryl poly (ethylene glycol 1000) succinate (TPGS) as biocompatible nanoprobes by thin film hydration method. Intravenous administration was carried out by tail vein injection of 200 μL (0.06 mmol) nanoprobes. Images at each channel were recorded at 1 min post-injection under the experimental condition (808 nm and 980 nm excitation, power density on the surface of mice was set to 175~200 mW/cm^2^, and exposure time was set to 100 ms). The images were finally processed with ImageJ software to get the ratiometic images.

Animal experiments were conducted in accordance with the guidelines of the Regional Ethics Committee for Animal Experiments and were approved by the Institutional Animal Care and Use Committee of Jilin University. The BALB/c mice were purchased from Liaoning Changsheng Biotechnology Co. Ltd. The license number is SCXK(Liao) 2020-0001.

1. **Theoretical Modeling of power dependent downshifting luminescence intensity**

The rate equation analysis provides a clear physical interpretation to identify further the dependence between downshifting luminescence and excitation power density. Generally, luminescence mechanisms in lanthanide systems with multiple electronic excited states include several processes, including ground state absorption (GSA), energy transfer (ET), back energy transfer (BET), luminescence, and nonirradiation relaxation (multiphonon relaxation and cross-relaxation). According to the spectra analyzed above and energy level diagrams of Tm^3+^, possible downshifting mechanisms are schematically illustrated in Figure S1, and we assume the possible model:

1) The ground-state population T_0_ density is constant.

2) The T_4_ excited state of Tm^3+^ is pumped from GSA of T_0_→T_4_ transition, and its population is determined by the absorption ($\sigma$) and the excitation power density ($\rho$).

3) The reaction steps between Yb^3+^ and subsequent excited states of Er^3+^ take place by ET and BET, and the ET and ETU rates are $\omega$ and $\omega_{b}$, respectively.

4) The excited states $i$ of Tm^3+^ have lifetimes $\tau_{i}$ and decay with rate constants$\alpha_{i}+\beta_{i}=\tau_{i}$, $\alpha_{i}$, $\beta_{i}$ represent radiative and NR rates, respectively.

5) The excited states $j$ of Yb^3+^ have lifetimes $\tau_{j}$ and decay with rate constants$\xi_{j}+\nu_{j}=\tau_{j}$, $\xi_{j}$, $\nu_{j}$ represent radiative and NR rates, respectively.


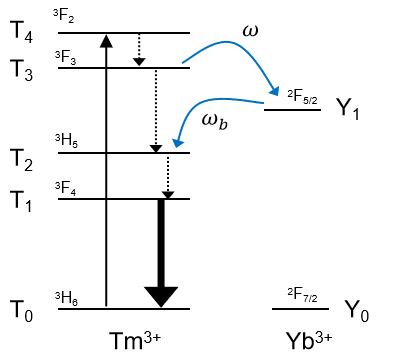


Taking into account the seven-level system schematically depicted in Figure S1, the rate equations that include all the energy transfer processes described above are given by

$$\frac{dY_{1}}{dt}=\omega T_{3}-\left( \xi_{1}+\nu_{1}+\omega_{b} \right)T_{1}=0 S1$$

$$\frac{dT_{1}}{dt}=\beta_{2}T_{2}-\left( \alpha_{1}+\beta_{1} \right)T_{1}=0 S2$$

$$\frac{dT_{2}}{dt}=\beta_{3}T_{3}+\omega_{b}T_{3}-\left( \alpha_{2}+\beta_{2} \right)T_{2}=0 S3$$

$$\frac{dT_{3}}{dt}=\beta_{4}T_{4}-\left( \alpha_{3}+\beta_{3}+\omega\right)T_{3}=0 S4$$

$$\frac{dT_{4}}{dt}=\sigma\rho T_{0}-\left( \alpha_{4}+\beta_{4} \right)T_{4}=0 S5$$

Thus,

$$T_{3}=\frac{\sigma\rho}{\left( \alpha_{3}+\beta_{3}+\omega\right)\left( \alpha_{4}+\beta_{4} \right)}\beta_{4}T_{0} S6$$

and the emission intensity $I$ of ^3^F_4_ level is given by

$${I=\alpha_{1}T}_{1}=\frac{\alpha_{1}\beta_{2}}{\left( \alpha_{1}+\beta_{1} \right)\left( \alpha_{2}+\beta_{2} \right)}\left[ \beta_{3}+\frac{\omega\omega_{b}}{\xi_{1}+\upsilon_{1}+\omega_{b}} \right]T_{3} S7$$

Theoretically, according to S6 and S7, we can get that $T_{3}\propto\rho,$ and $I\propto\rho$. That is, the luminescence from ^3^F_4_ (Tm^3+^) is proportional to the excitation power density.

If Yb^3+^ is absent in the system, we can obtain the rate equations:

$$\frac{dT_{1}}{dt}=\beta_{2}T_{2}-\left( \alpha_{1}+\beta_{1} \right)T_{1}=0 S8$$

$$\frac{dT_{2}}{dt}=\beta_{3}T_{3}-\left( \alpha_{2}+\beta_{2} \right)T_{2}=0 S9$$

$$\frac{dT_{3}}{dt}=\beta_{4}T_{4}-\left( \alpha_{3}+\beta_{3} \right)T_{3}=0 S10$$

$$\frac{dT_{4}}{dt}=\sigma\rho T_{0}-\left( \alpha_{4}+\beta_{4} \right)T_{4}=0 S11$$

Thus, the emission intensity is given by

$${I'=\alpha_{1}T}_{1}=\frac{\alpha_{1}\beta_{2}}{\left( \alpha_{1}+\beta_{1} \right)\left( \alpha_{2}+\beta_{2} \right)}\beta_{3}T_{3} S12$$

According to S12, we can find that the luminescence from ^3^F_4_ is also proportional to the excitation power density. However, according to S7 and S12, The enhancement part of the population of ^3^F_4_ level with Yb^3+^ doping is determined by the energy transfer rate (*ω*) and back energy transfer rate (*ω_b_*) between Tm^3+^ and Yb^3+^:

$$\frac{\alpha_{1}\beta_{2}}{\left( \alpha_{1}+\beta_{1} \right)\left( \alpha_{2}+\beta_{2} \right)}\cdot\frac{\omega\omega_{b}}{\xi_{1}+\upsilon_{1}+\omega_{b}}T_{3}$$

1. **Fluorescence intensity ratio analysis**

The fluorescence intensity ratio of ONNPs under fixed irradiation power density of 808 and 980 nm excitation is calculated by:

$$R=\frac{I_{1825}}{I_{1530}}=\frac{P_{808}{\cdot e^{-\alpha_{808}l}\cdot A}_{808}{\cdot\eta}_{1825}{\cdot e}^{-\alpha_{1825}l}}{P_{980}{\cdot e^{-\alpha_{980}l}\cdot A}_{980}{\cdot\eta}_{1530}{\cdot e}^{-\alpha_{1530}l}}$$

$P_{808}$ and $P_{980}$ represent the power density of 808 and 980 nm irradiation; $\alpha_{i}$ (i=808, 980, 1530 and 1825) are optical attenuation of the bio-tissue at 808, 980, 1530 and 1825 nm, respectively; *l* is the depth of ONNPs in the bio-tissue; and $A_{i}$ denoted the absorption cross-section of ONNPs; and $\eta_{i}$ denoted the photon quantum yield of ONNPs at different emission bands. Because *P*, A, and $\eta_{i}$ are fixed, the R can be obtained from:

$$R=\frac{I_{1825}}{I_{1530}}=C\cdot e^{-(\alpha_{808}-\alpha_{980})l}\cdot e^{-(\alpha_{1825}-\alpha_{1530})l}$$

That is to say, the relationship of the fluorescence intensity ratio to the depth is exponential:

$$R=C\cdot e^{-\alpha l}$$

Or it can be expressed as

$$\ln R=C-[{\Delta\alpha}_{1}+{\Delta\alpha}_{2}]l$$

${\Delta\alpha}_{1}$ and ${\Delta\alpha}_{2}$ represent the different optical attenuation of excitation wavelength and emission wavelength, respectively.

1. **Data**


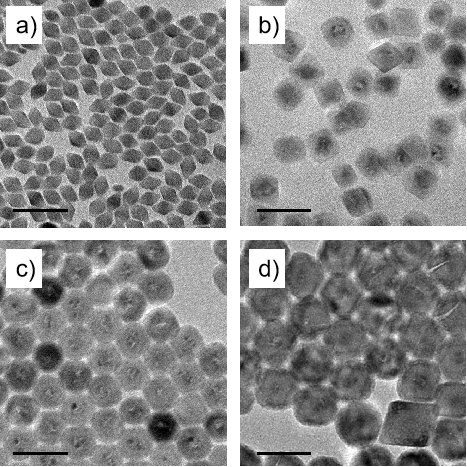


**Figure S1.** TEM images of a)LiTmF_4_: 5% Yb; b)LiTmF_4_: 5% Yb@LiYF_4_; c) LiTmF_4_: 5% Yb@LiYF_4_@LiYbF_4_: 2% Er; d) LiTmF_4_: 5% Yb@LiYF_4_@LiYbF_4_: 2% Er@LiYF_4_ nanoparticles. Scale bar in images: 50 nm.


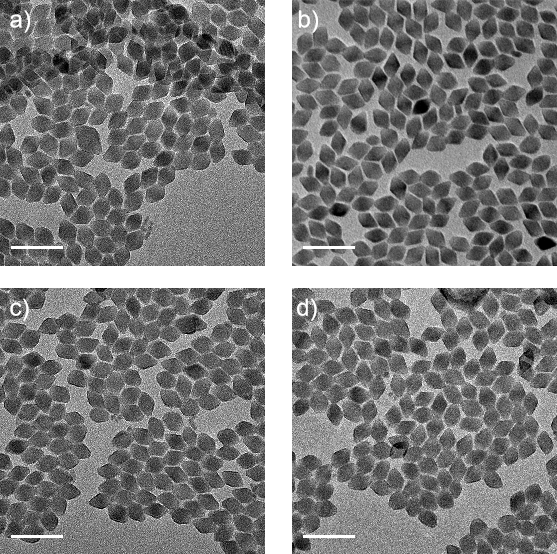


**Figure S2.** TEM images of LiTmF_4_: x% Yb@LiYF_4_ core@shell nanoparticles:

a) x=0; b) x=5; c) x=10; d) x=20. Scale bar in images: 50 nm.


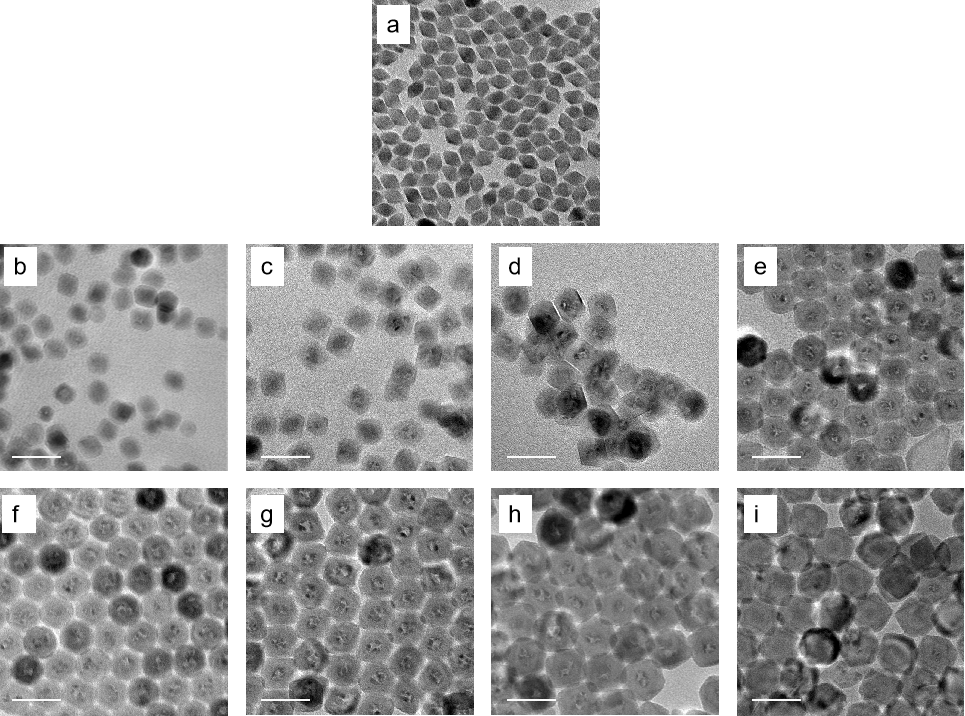


**Figure S3**. TEM images of (a) LiTmF_4_:5%Yb core NPs and (b-e) LiTmF_4_:5%Yb@LiYF_4_ core-shell NPs with different LiYF_4_ shell thickness: (b) 1.5 nm, (c) 5.0 nm, (d) 6.5 nm, and (e) 8.0 nm. (f-g) TEM images of corresponding LiTmF_4_:5%Yb@LiYF_4_@LiYbF_4_:2%Er@LiYF_4_ core@shell nanoparticles with same LiYbF_4_:2%Er and LiYF_4_ shell thicknesses. Scale bar in images: 50 nm.


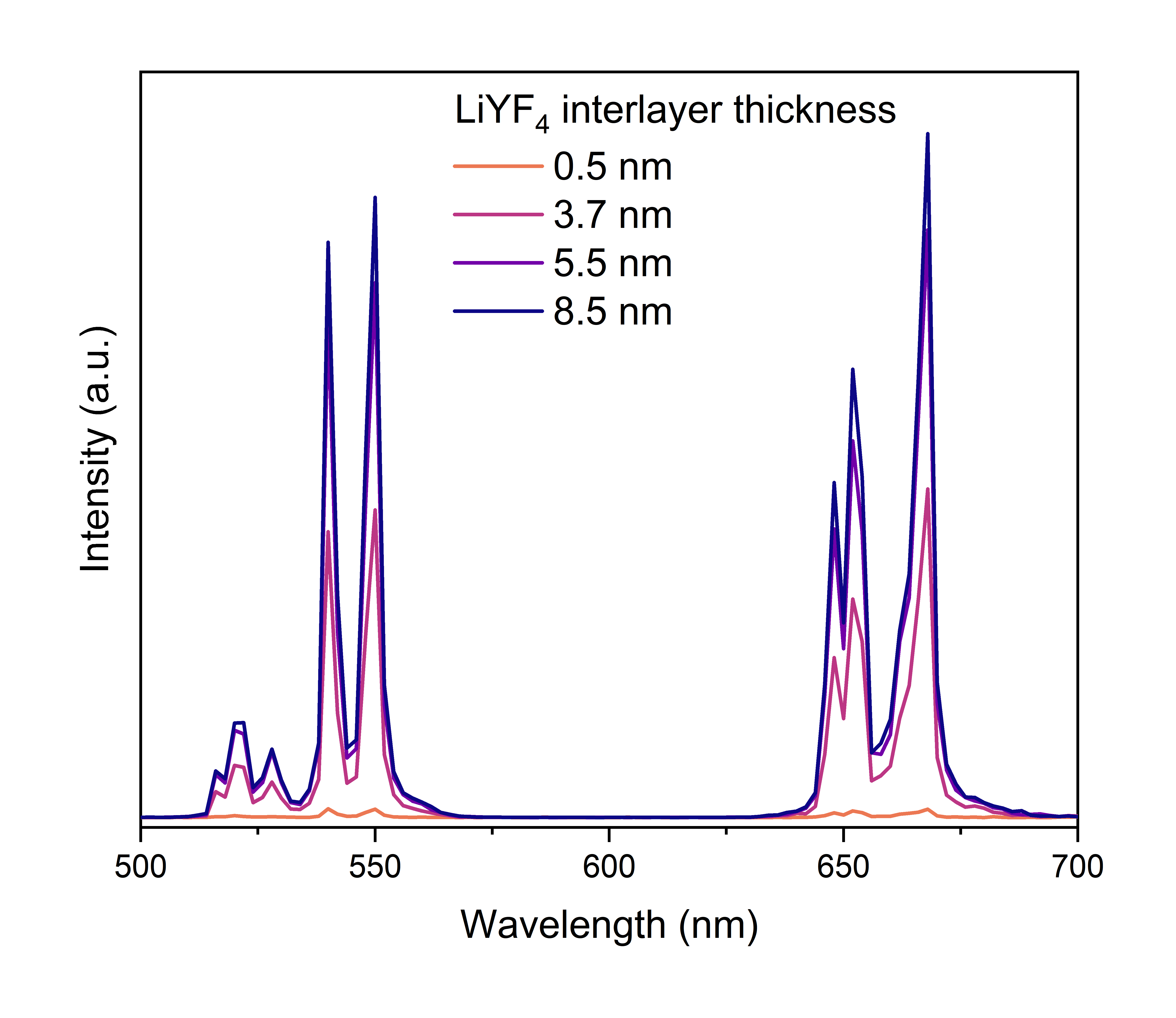


**Figure S4.** Upconversion emission spectra of ONNPs with a different thickness of LiYF_4_ interlayer under 980 nm excitations.


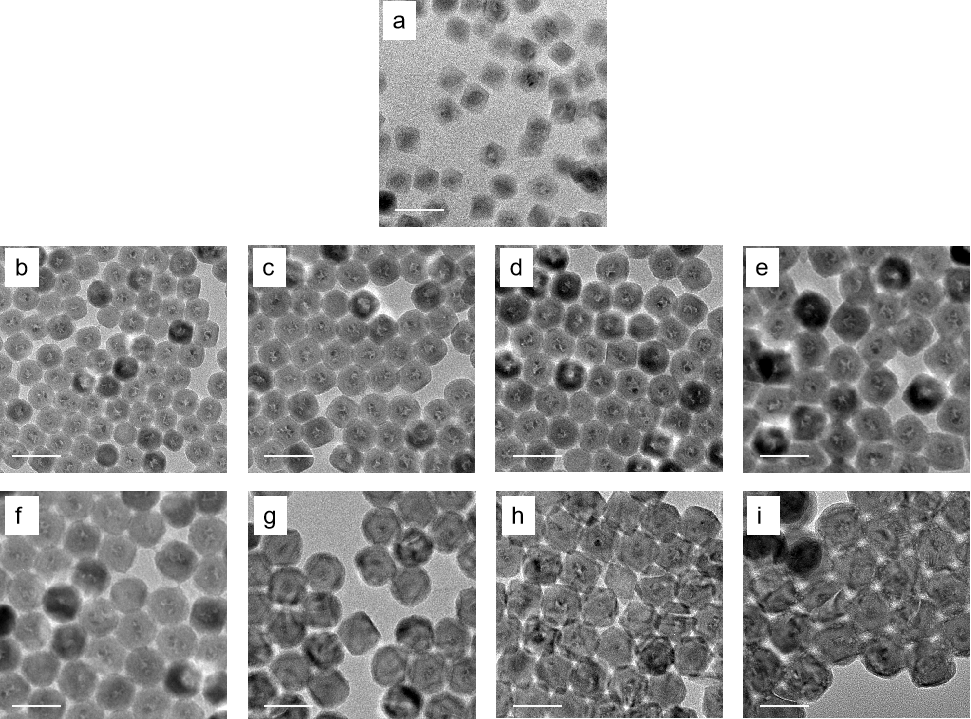


**Figure S5.** TEM images of (a) LiTmF_4_:5%Yb@LiYF_4_, (b-e) LiTmF_4_:5%Yb@LiYF_4_@LiYbF_4_:2%Er NPs with different LiYbF_4_:2%Er shell thicknesses: (b) 1 nm, (c) 1.7 nm, (d) 3.0 nm and (e) 5.2 nm. (f-g) TEM images of corresponding LiTmF_4_:5%Yb@LiYF_4_@LiYbF_4_:2%Er@LiYF_4_ core@shell NPs with the same LiYF_4_ shell thickness. Scale bar in images: 50 nm.


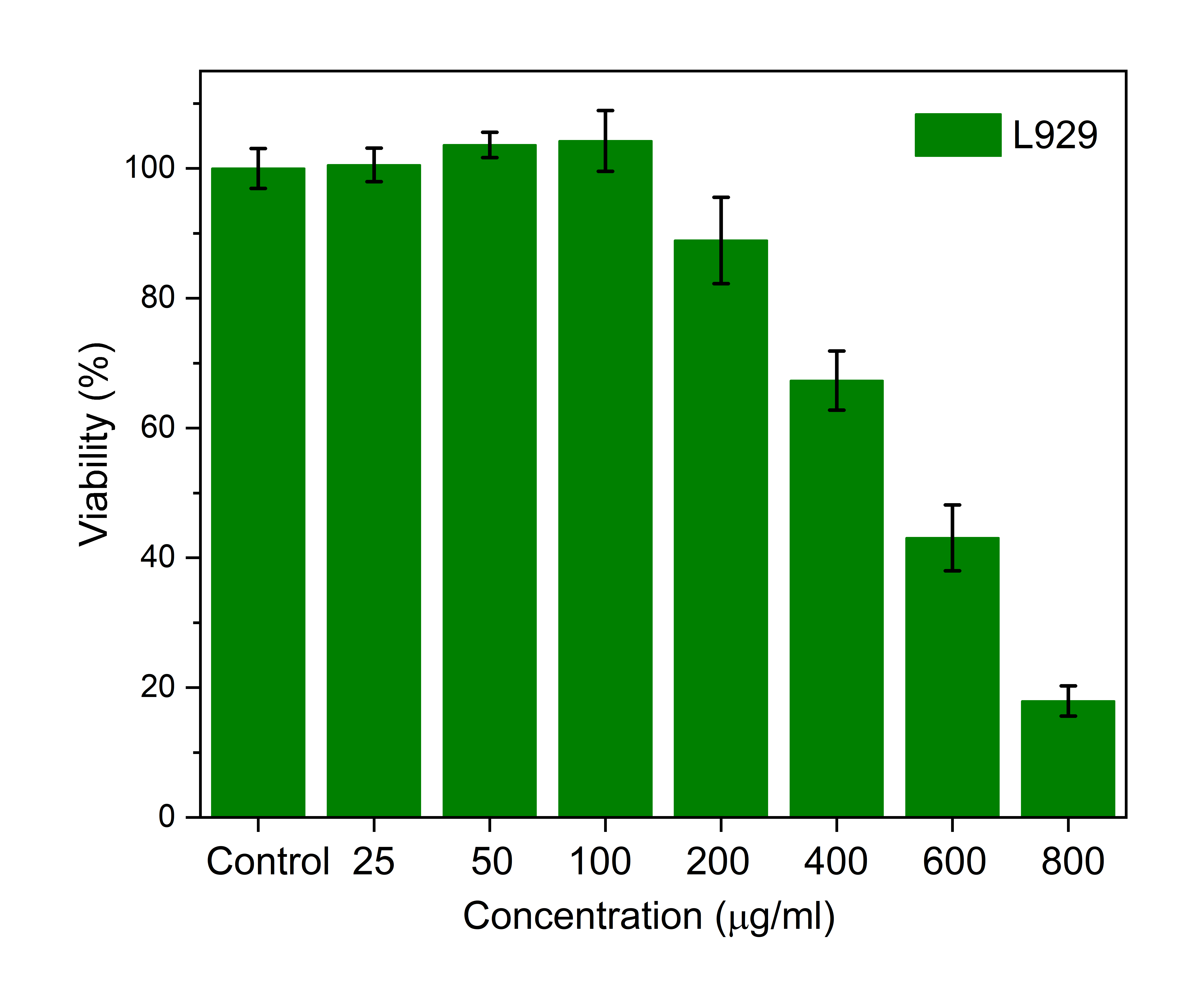


**Figure S6.** Cell viabilities of L929 cells (mouse fibroblasts cells) at different concentrations of ONNPs.


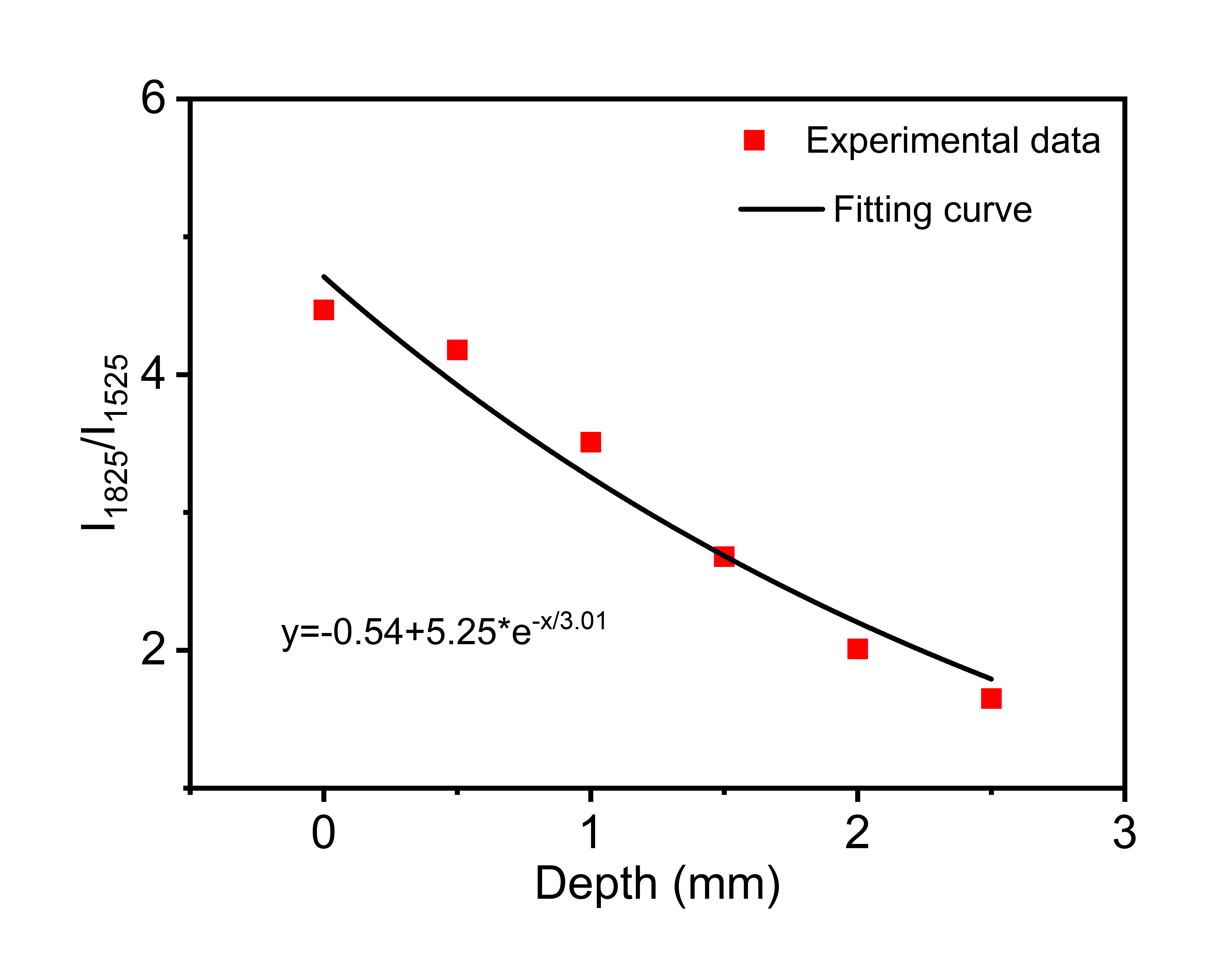


**Figure S7.** The experimental relationship of fluorescence intensity ratio to the depth of ONNP@TPGS in 1% intralipid solution.


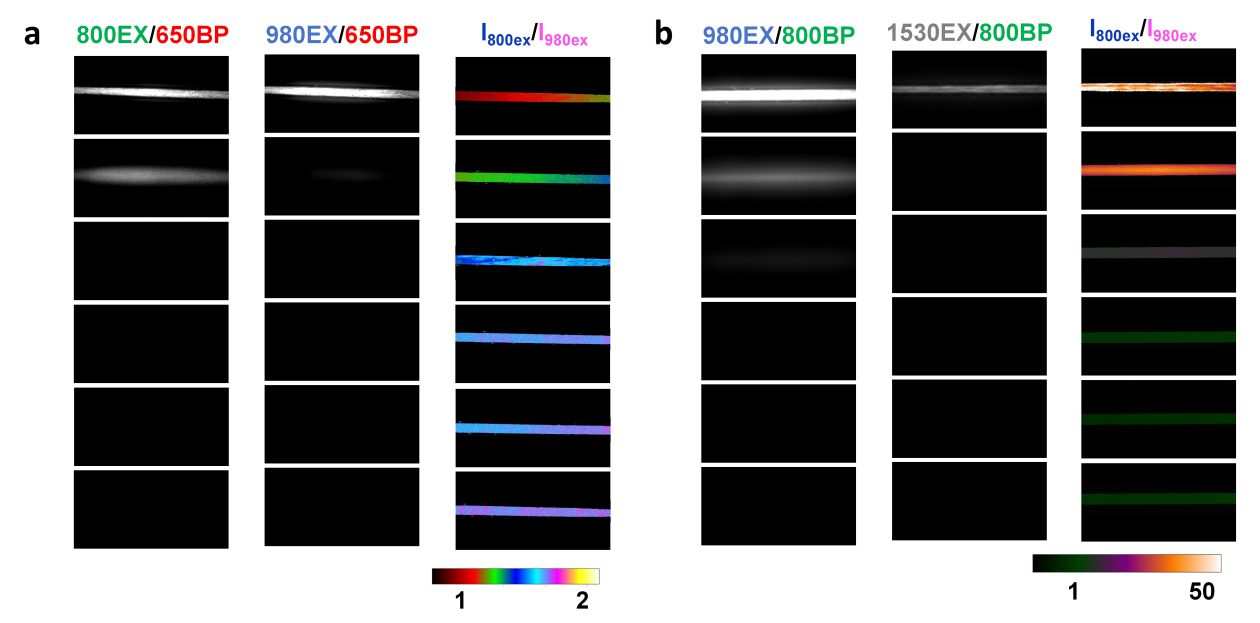


**Figure S8.** a) Visible and b) NIR-I luminescence and corresponding ratiometric images of a capillary containing NaErF_4_@NaYbF_4_@NaYF_4_-based probe covered with different thicknesses of 1 wt% intralipid under 808/980 nm excitation and 980/1530 nm excitation, respectively.

<bib id="bib1" type="Periodical"><number>[1]</number>X. Y. Xie, Q. Q. Li, H. R. Chen, W. Wang, F. X. Wu, L. P. Tu, Y. L. Zhang, X. G. Kong, Y. L. Chang, *Nano Lett.* **2022**, *22*, 5339.[[CrossRef](http://dx.doi.org/10.1021/acs.nanolett.2c01324)]</bib>
